# DRUG INTOXICATION AMONG LATINO OPIOID USERS IN FLORIDA

**DOI:** 10.1093/geroni/igae098.1584

**Published:** 2024-12-31

**Authors:** Armiel Suriaga, Ruth Tappen, Raquel Panos, Christina Dimeo

**Affiliations:** Florida Atlantic University, Boca Raton, Florida, United States; Florida Atlantic University, Boca Raton, Florida, United States; Florida Atlantic University, Boca Raton, Florida, United States; Florida Atlantic University, Boca Raton, Florida, United States

## Abstract

The opioid epidemic remains a serious public health problem, killing > half a million people since 1999, including racial/ethnic groups, i.e., Hispanics. However, Drug Intoxication (DI) from opioid use, particularly in Florida, where the Hispanic population totaled 5.469 million in 2020, is seldom reported. We aimed to report the characteristics of Hispanic decedents with DI, particularly opioids and other substances, from 2020 to 2021. We also examined the association of being Hispanic to DI and the co-use of opioids, cannabis, and alcohol. We used descriptive statistics and regression models to analyze de-identified data from the Florida Department of Law Enforcement using Stata 17. 1,199 Hispanic decedents out of 30,846 people who died from any drugs were analyzed. Mean age=39.14 (SD 13.89); males (n=1,015 or 84.7%) between the 25-34 age group (n=339 or 34.9%), followed by the 35-44 age group (n= 306 or 25.5%); only a small proportion of older Hispanic decedents (n=58 or 4.8%). 551 out of 1,199 Hispanic decedents died from DI. Being Hispanic was not significantly associated with DI (p=.053). In the intercept model, being Hispanic has 0.87 odds of dying from DI (OR=.87, [95% confidence interval=.77-.98], p=0.018. After adjusting for confounders (age, gender, population density, and other substances, the odds of dying from DI slightly increased (OR=.89, [95% CI =.76-1.03], p=0.117; adding clinical covariates (cardiac, diabetes, kidney, and liver-related problems) OR=.85, 95% CI=.74-.99], p=0.038. Among opioid users (OR=16.46, 95% CI=15.47-17.50], p=<0.001. Public safety necessitates more research to elucidate factors driving DI among this ethnic minority.

